# Tungiasis (jigger infestation) in Rural Kenya, an emerging infectious disease

**DOI:** 10.1186/1742-4690-9-S1-P37

**Published:** 2012-05-25

**Authors:** Nicholas N Njau, Peter Wanzala, Marion Mutugi, Liana Ariza, Jorg Heukelbach

**Affiliations:** 1 Kenya Medical Research Institute, Center for Public Health Resear, Nairobi, Kenya

## Objective

To describe the prevalence of tungiasis (jigger flea infestation) and associated risk factors in a sentinel group (children 5-12 years of age) in rural Central Kenya.

## Methods

A cross-sectional study was carried out in Murang’a South district during high transmission season (dry season, August – September 2009). A total of 385 randomly selected households were visited. Children were examined for presence of tungiasis, and a questionnaire was administered to collect demographic, behavioral and environmental data.

## Results

Prevalence of tungiasis was 57% (218/385; 95% CI=51.7%-61.6%). Itching (89.1%) was the most common associated symptom, followed by pain upon pressure (67.3%), sleep disturbance (58.2%) and walking difficulties (53%). In multivariate logistic regression analysis the following independent factors were identified to be associated with tungiasis: living in houses with an earthen floors (adjusted OR=3.84; 95%IC: 2.09-7.06), walking barefooted (OR=3.28; 1.78-6.04), having a common resting place outside the house (OR=2.36; 1.01-5.51) and presence of rats on the compound (OR=1.69; 1.03-2.75).

## Conclusion

Tungiasis is an emerging neglected disease found in Africa. It is highly endemic in rural Central Kenya and associated with considerable morbidity. The disease is associated with poverty. Modifiable risk factors were identified that should be the focus of sustainable and effective control measures.

